# Deep Network Pharmacology: Targeting Glutamate Systems as Integrative Treatments for Jump-Starting Neural Networks and Recovery Trajectories

**DOI:** 10.20900/jpbs.20210008

**Published:** 2021-04-30

**Authors:** R. Andrew Chambers, Christopher Toombs

**Affiliations:** 1 Lab for Translational Neuroscience on Dual Diagnosis, IU Neuroscience Research Center, 320 West 15th Street, Indianapolis, IN 46202, USA; 2 Proniras Corp, Seattle, WA 98102, USA

**Keywords:** addiction, mental illness, deep network pharmacology, integrative treatments, glutamate

## Abstract

Significant advances in pharmacological treatments for mental illness and addiction will require abandoning old monoaminergic theories of psychiatric disorders and traditionally narrow approaches to how we conduct treatment research. Reframing our efforts with a view on integrative treatments that target core neural network function and plasticity may provide new approaches for lifting patients out of chronic psychiatric symptom sets and addiction. For example, we discuss new treatments that target brain glutamate systems at key transition points within longitudinal courses of care that integrate several treatment modalities. A reconsideration of what our novel and already available medications are intended to achieve and how and when we deliver them for patients with complex illness trajectories could be the key to unlocking new advances in general and addiction psychiatry.

Addiction and mental illnesses are physiologically convergent and causally inter-related diseases of the brain that involve disturbances in decision making, motivation, cognition, stress-responsivity, and social behavior [1,2]. Unfortunately, progress in the discovery and delivery of new treatment modalities for brain-behavioral health has slowed considerably in recent years despite ongoing investments in basic and clinical neuropsychiatric research.

Much needed reentry into a stream of new advances will require not only more research but new research perspectives that challenge and strive beyond traditional views. Long held uni-transmitter theories of mental illness and addictive disorders have perpetuated a focus on the usual monoamine systems. This aging dogma is no longer advancing psychiatric care beyond introducing ‘me-too’ drugs that lack significant innovation. At the same time, the brain is not merely a container of a small number of neurotransmitters that just need to be corrected to the proper levels. Rather, it is a vast, incredibly complex, and highly plastic neural network. Many modulatory neurotransmitter systems and excitatory/inhibitory sub-circuits undergo neurodevelopmental revision through fetal development, adolescence and adulthood. Through this development, they can be and often are adversely impacted by maladaptive re-modelling, producing both psychiatric and substance use disorders [3]. Moreover, these networks are developmentally and experientially sculpted, not only based on an individual’s proscribed genetic makeup, but also by one’s life narrative of complex psychosocial, stress-ridden, motivational and contextual learning experiences. Entertaining the perspective of a neural network lends greater sophistication in contemplating therapeutic interventions, not only by traditional means such as augmenting neurotransmitter levels or antagonizing receptor engagement, but more so as about altering the structure, function, and plasticity of the neural network, to achieve new functional states [4].

This ‘neural network-as-therapeutic-target’ viewpoint pries open the door to understanding the need for more aggressive pursuit of *integrative treatments* in brain-behavioral-health. By ‘integrative’, the approach advocated here is twofold.

## First, by integrative treatments, we are referring to the concurrent integration of two or more different treatments to target one single diagnosis.

Pharmacologically speaking, this is conceptually similar to medical practice in oncology or infectious disease where two or more drugs are utilized in combination to more formidably attack a neoplasm or pathogen. The corollary is also evident in evidence-based psychiatric practices which, for example, augment SSRI medications with lithium or atypical neuroleptics in treatment-refractory mood disorders. Yet, concretely established research on optimal combined medication approaches is still largely lacking, even as complex polypharmacy regimens that have little empirical support have become commonplace in psychiatric care.

Another form of integrated treatment that is perhaps even more richly informed by neural network theory, is the integration of medication and experiential-psychotherapy treatments. If we intend to develop new medication regimens that more aggressively alter neural network architecture and plasticity more profoundly than traditional medications, then we should be researching how the environment and psychosocial-experiences of the patient can be therapeutically optimized during (or in the aftermath of) pharmacologic treatment to ensure better outcomes. This concept attends to the neural network-informed idea that healthy brains work effectively as orchestrators of environmental-responsivity-and-adaptation where improved neural function generates a better mapping of behavior (and more satisfying experience) onto overcoming the challenges of an ever-changing environment. So, it is important to know how medication-assisted neural remodeling may be guided by psychotherapies and other experiences that also impact neural remodeling and successful environmental adaptation.

The research community is beginning to more seriously pursue treatment advances that may exceed older medications in terms of evoking more profound experiential effects and neuroplastic responses in the brain. Mounting evidence suggests that LSD/psilocybin, MDMA, ketamine (see these excellent reviews: [5–7]), and various neurostimulatory techniques (e.g., advanced rTMS and Direct Stimulation approaches [8–12]) are efficacious for various mental illnesses and addictions, potentially beyond what our current repertoire of standard FDA-approved approaches can deliver [13–16]. Still, there is an undeniable need for more studies that integrate medication, neurostimulatory and psychotherapeutic experiences as inspired by studies that are now many decades old, but were never adequately afforded an opportunity to develop into rigorously defined research campaigns [17–19]. Cutting edge treatment research now being explored in this vein combines the real-time, active participation of the patient in the treatment experience as made possible through neuroimaging-assisted biofeedback, self-directed neurostimulation, and even virtual reality experiences that may combine with medication and/or neurostimulation [20–26].

## The second meaning for integrative treatments, refers to the idea that one treatment modality is relevant to multiple diagnoses.

While there is a tradition for a somewhat parsimonious treatment approach in psychiatry (e.g., SSRIs are effective for treating both depression and anxiety disorders), the research community has not extended its efforts far enough, especially when it comes to looking for treatments that target both mental disorders and addictions. Therein lies a major gap in funded research that is clear in light of the fact that mental illnesses and addiction typically coexist within the same patient. These diseases involve shared/overlapping neurocircuits and shared causal factors spanning genetic and traumatic experiences [27].

Funding from the brain-behavioral health focused federal institutes (NIMH, NIAAA, NIDA) has not yet been substantially responsive to the need for integrative treatments on the psychotherapy/medication or mental health/addiction interfaces. However, there is growing federal support for new drug development that advances us beyond ages-old monoamine pathways and even traditional modalities of drug delivery. For instance, consider brexanolone, the novel treatment for post-partum depression. Brexanolone is a naturally occurring neurosteroid (allopregnanolone) that is a positive allosteric modulator of GABAA receptors [28]. Also, consider ketamine (and its FDA-approved enantiomer esketamine), as another novel treatment for depression that interacts with the glutamate system as an NMDA-receptor antagonist [13]. Besides representing a couple of the most truly innovative treatment developments in the last 25 years, these medications share some other interesting attributes: (1) they act much more rapidly than standard medications; (2) they are not delivered orally (thus requiring medically supervised administration); and (3) their pharmacologic effects involves GABA and/or glutamate systems. While getting us beyond targeting monoamine transmission, the latter attributes are especially interesting because GABA and glutamate neurotransmission are core mediators of information flow and neuroplasticity within neural networks that are involved in both mental illness and addiction. In other words, these treatments may also represent potential *integrative treatment* modalities.

At face value, the shared attributes of these treatments—that they cannot be delivered by an oral route, or outside of professional monitoring and supervision—could be considered as major limitations. However, accepting and leveraging these attributes may be important for making many more real advances in psychopharmacology. The premise of treatment delivery occurring under medical supervision adds assurance of greater safety, especially if new agents, as a correlate of their capacity for invoking deep neuroplastic effects, also have more acute psychoactive/intoxicating effects. Moreover, this supervision also provides a time window and clinical context for delivery of psychotherapies or therapeutic experiences *concurrent* with the medication delivery or neurostimulation.

**Figure 1.**
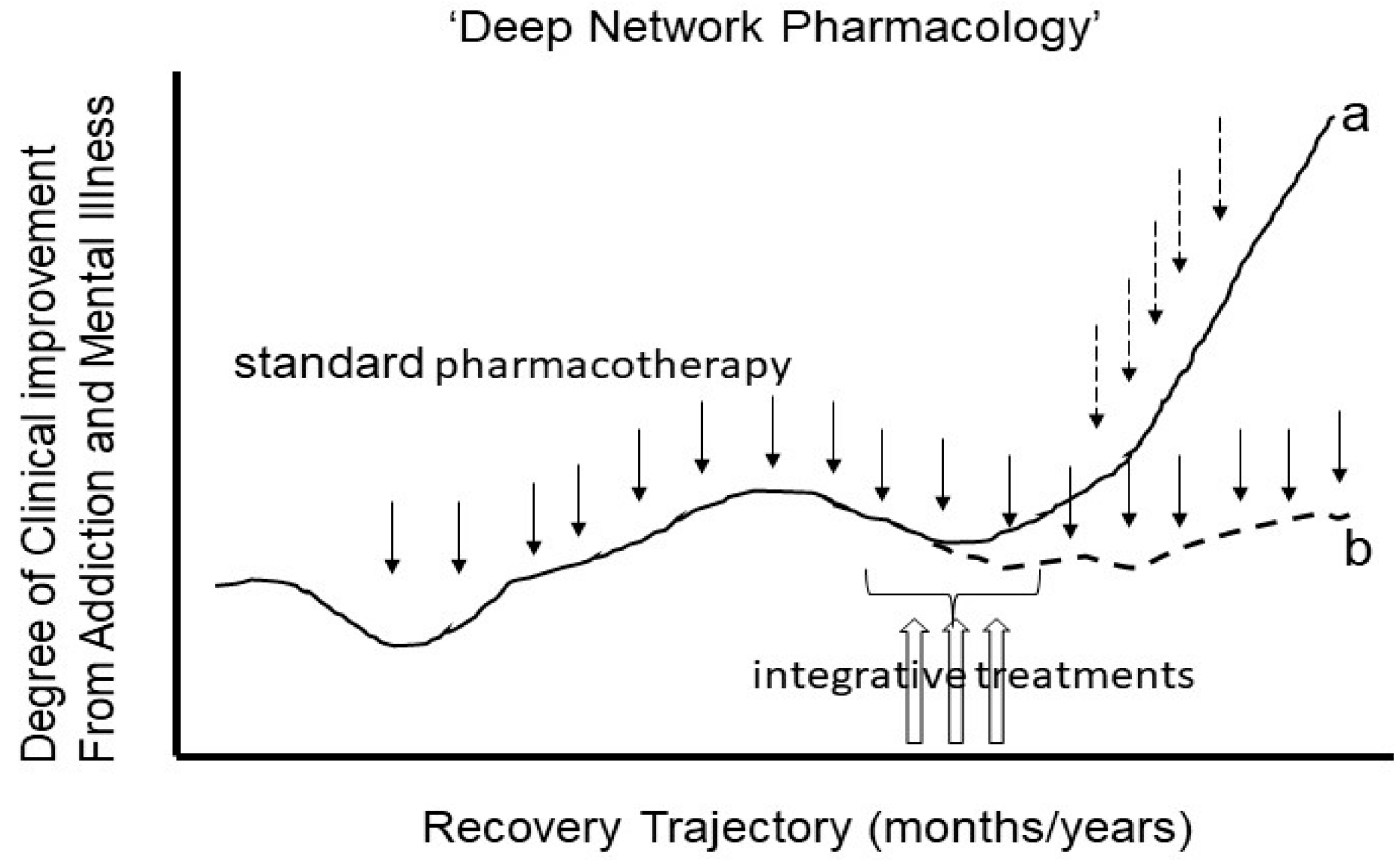
A new frontier of research on ‘Deep Network Pharmacology’ seeks to apply integrative treatments at critical junctures of patient’s recovery trajectories. Whereas standard pharmacotherapies focused on monoamine systems or other modulatory receptor systems (DA, 5-HT, NE, mu-opioid) may produce partial improvements in illness course (fine downward solid arrows), many patients, especially those with complex comorbidities of addiction and mental illness, can become mired in a local minimum where, even with continued standard medication maintenance and good compliance, the brain cannot change sufficiently to allow further significant recovery (trajectory b). However, the relatively short-term application of more intensive integrative treatments (3 upward unfilled arrows), e.g., targeting glutamate neurotransmission networks within these local minima, may more profoundly invoke neuroplastic change in conjunction with proximal psychotherapeutic experiences. This Deep Network pharmacology approach may help patients gain momentum into new recovery trajectories (trajectory a) with continued standard meds and/or as part of a transition to a different medication/psychotherapy combination (fine downward stippled arrows).

Thus, we advocate for intensive neural network-informed integrative treatments that may be employed at key phases of a patient’s outpatient addiction/mental health treatment plan, in a way that is not disconnected from, but is responsive to their position on their overall illness-to-recovery trajectory. We can expect that highly integrative treatments—what we might also call a new frontier of ‘Deep Network Pharmacology’—should aim to intensively invoke network plasticity, to help unshackle brain function from a ‘local minimum state’ where they may have become chronically mired, providing new momentum toward a better recovery trajectory (Figure 1).

In the forum of addiction treatments, NIDA is broadcasting research themes in keeping with these ideas as indicated by publishing a statement on their ‘10 most wanted’ target mechanisms [29]. In this list, a range of innovative treatments targeting brain systems implicated in both mental illness and addiction (many of them involving GABA or glutamate neurotransmission; and some that involve other than oral administration) are being prioritized. In pursuit of one of the ‘10 most wanted’, our research team, accompanied by NIDA support, and seminal basic science performed nearly a quarter century ago [30] is exploring the utility of a glutamate-AMPA/kainate receptor antagonist (tezampanel) delivered intravenously. This drug, which also confers protection against neurotoxic events, shows potential for enhancing successful transition of patients from opioids, through withdrawal, to non-opioid-based long-term pharmacotherapies for opioid addiction. This potentially highly integrative treatment modality might work concurrent with other treatments for opioid overdose, opioid withdrawal or addiction. Also, tezampanel may have implications for other forms of withdrawal, other types of addiction, and other mental illnesses. With our research eyes wide open to the ‘target rich’ environment present in the brain, and the perspective that neural network-informed integrative treatment pursuit gives us, we are more likely to be successful in the discovery of advanced therapeutics in the years to come.
